# Supplementary material for: A UMLS-based spell checker for natural language processing in vaccine safety
Source: BMC Med Inform Decis Mak. 2007 Feb 12;7:3. doi: 10.1186/1472-6947-7-3 (PMC1805499; doi:10.1186/1472-6947-7-3)
Supplement: Additional file 13 — RAP application directory. Sets up directory for RAP (RDF application for PHP) source code [file 1472-6947-7-3-S13.gz › rap/doc/phpdoc/syntax/GRDDLParser.html]

xml version="1.0" encoding="iso-8859-1"?


Docs For Class GRDDLParser


## Class GRDDLParser

Description

Description |
Methods (details)

A GRDDLParser.

This class extracts rdf data from xhtml documents. It uses the PHP xsltprocessor. Gleaning Resource Descriptions from Dialects of Languages (GRDDL): (http://www.w3.org/TR/grddl/)

History:

- 10-26-2004 : first version

- access: public
- author: Tobias Gau� <tobias.gauss@web.de>,
- version: V0.9.1

Located in /syntax/GRDDLParser.php (line 24)

```
Object
   |
   --GRDDLParser
```

Method Summary

Description |
Methods (details)

MemModel
generateModel
(String $doc)

void
\_generateRDF
()

Methods

Description |
Methods (details)

generateModel (line 63)

generates a MemModel and creates the DomDocument.

- access: public

MemModel

generateModel
(String $doc)

- String
  $doc

\_generateRDF (line 106)

void

\_generateRDF
()

#### Inherited Methods


Inherited From Object

> Object::toString()

Documentation generated on Thu, 7 Jul 2005 13:41:28 +0200 by phpDocumentor 1.3.0RC3
